# Factors Related to Increasing Prevalence of Resistance to Ciprofloxacin and Other Antimicrobial Drugs in *Neisseria gonorrhoeae*, United States

**DOI:** 10.3201/eid1808.111202

**Published:** 2012-08

**Authors:** Edward Goldstein, Robert D. Kirkcaldy, David Reshef, Stuart Berman, Hillard Weinstock, Pardis Sabeti, Carlos Del Rio, Geraldine Hall, Edward W. Hook, Marc Lipsitch

**Affiliations:** Harvard School of Public Health, Boston, Massachusetts, USA (E. Goldstein, M. Lipsitch);; Centers for Disease Control and Prevention, Atlanta, Georgia, USA (R.D. Kirkcaldy, S. Berman, H. Weinstock);; Oxford University, Oxford, UK (D. Reshef);; Harvard University, Cambridge, Massachusetts, USA (P. Sabeti);; Emory University, Atlanta (C. Del Rio);; Cleveland Clinic, Cleveland, Ohio, USA (G. Hall); University of Alabama at Birmingham, Birmingham, Alabama, USA (E.W. Hook);; and Jefferson County Department of Health, Birmingham (E.W. Hook)

**Keywords:** Neisseria gonorrhoeae, antimicrobial resistance, men who have sex with men, multidrug resistance, MSM, bacteria, gonorrhea, United States, ciproflaxin, luoroquinolones

## Abstract

Using data from the Gonococcal Isolate Surveillance Project, we studied changes in ciprofloxacin resistance in *Neisseria gonorrhoeae* isolates in the United States during 2002–2007. Compared with prevalence in heterosexual men, prevalence of ciprofloxacin-resistant *N. gonorrhoeae* infections showed a more pronounced increase in men who have sex with men (MSM), particularly through an increase in prevalence of strains also resistant to tetracycline and penicillin. Moreover, that multidrug resistance profile among MSM was negatively associated with recent travel. Across the surveillance project sites, first appearance of ciprofloxacin resistance in heterosexual men was positively correlated with such resistance for MSM. The increase in prevalence of ciprofloxacin resistance may have been facilitated by use of fluoroquinolones for treating gonorrhea and other conditions. The prominence of multidrug resistance suggests that using other classes of antimicrobial drugs for purposes other than treating gonorrhea helped increase the prevalence of ciprofloxacin-resistant strains that are also resistant to those drugs.

## Abstract 2

What would you do if you had a sexually transmitted disease that was untreatable with antibiotics? That is the situation we may be heading toward. In the United States, gonorrhea is the second most common reportable infection. Over the years, the organism that causes it, *N. gonorrhoeae*, has acquired resistance to several classes of antibiotics including, most recently, the fluoroquinolones. In fact, widespread resistance led CDC to stop recommending fluoroquinolones for gonorrhea treatment in 2007. Today, cephalosporin-based combination therapy is the last remaining option currently recommended for gonorrhea treatment. Understanding of the causes of drug resistance is needed so that control measures can be improved and the effectiveness of the few remaining drugs can be maintained. This article investigates possible causes for the emergence of fluoroquinolone-resistant *N. gonorrhoeae* that occurred several years ago. Fluoroquinolone-resistant strains spread in the United States in the late 1990s and spread more rapidly among men who have sex with men (MSM) than among heterosexual men. One possible explanation for the rise in drug resistance, especially among heterosexuals, is acquisition of resistant gonorrhea through travel. Certain drug-resistant strains of *N. gonorrhoeae*, particularly the multidrug resistant strains (also resistant to penicillin and tetracycline) circulating among MSM, seemed to be able to reach high prevalence levels through domestic transmission, rather than through frequent importation. After resistance emerged in a geographic area, resistant strains appeared among MSM and heterosexuals within several months. When resistance is detected in either MSM or heterosexuals, prevention efforts should be directed toward both populations.

## Medscape CME Activity

Medscape, LLC is pleased to provide online continuing medical education (CME) for this journal article, allowing clinicians the opportunity to earn CME credit.

This activity has been planned and implemented in accordance with the Essential Areas and policies of the Accreditation Council for Continuing Medical Education through the joint sponsorship of Medscape, LLC and Emerging Infectious Diseases. Medscape, LLC is accredited by the ACCME to provide continuing medical education for physicians.

Medscape, LLC designates this Journal-based CME activity for a maximum of 1 *AMA PRA Category 1 Credit(s)*^TM^. Physicians should claim only the credit commensurate with the extent of their participation in the activity.

All other clinicians completing this activity will be issued a certificate of participation. To participate in this journal CME activity: (1) review the learning objectives and author disclosures; (2) study the education content; (3) take the post-test with a 70% minimum passing score and complete the evaluation at www.medscape.org/journal/eid; (4) view/print certificate.

**Release date: July 16, 2012; Expiration date: July 16, 2013**

## Learning Objectives

Upon completion of this activity, participants will be able to:

- Describe overall patterns of drug resistance stratified by sexual orientation, based on an analysis of data from GISP
- Describe the association of recent travel with drug resistance in MSM and heterosexuals, based on an analysis of data from GISP
- Describe the first appearance of drug resistance in heterosexuals and MSM, based on an analysis of data from GISP.

## CME Editor

**Carol E. Snarey, MA,** Technical Writer/Editor, *Emerging Infectious Diseases. Disclosure: Carol E. Snarey, MA, has disclosed no relevant financial relationships.*

## CME Author

**Laurie Barclay, MD,** freelance writer and reviewer, Medscape, LLC. *Disclosure: Laurie Barclay, MD, has disclosed no relevant financial relationships.*

## Authors

*Disclosures:* ***Edward Goldstein, PhD; Robert D. Kirkcaldy, MD, MPH; David Reshef; Stuart Berman, MD; Hillard Weinstock, MD, MPH; Pardis Sabeti, MD, DPhil; Geraldine Hall, PhD;*** *and* ***Marc Lipsitch, PhD,*** *have disclosed no relevant financial relationships.* ***Carlos Del Rio, MD,*** *has disclosed the following relevant financial relationships: served as an advisor or consultant for Gilead Sciences; received grants for clinical research from Merck and Co.* ***Edward W. Hook, MD,*** *has disclosed the following relevant financial relationships: served as an advisor or consultant for Cempra; served as a speaker or a member of a speakers bureau for Becton Dickinson; received grants for clinical research from Becton Dickinson, Cepheid, Roche Molecular, Gen-Probe Inc., Cempra, Siemens, GlaxoSmithKline; acted as a book editor and received royalties from McGraw Hill.*
