# Supplementary material for: Factors Related to Increasing Prevalence of Resistance to Ciprofloxacin and Other Antimicrobial Drugs in Neisseria gonorrhoeae, United States
Source: Emerg Infect Dis. 2012 Aug;18(8):1290–7. doi: 10.3201/eid1808.111202 (PMC3414012; doi:10.3201/eid1808.111202)
Supplement: Related texts

# Research article: Factors Related to Increasing Prevalence of Resistance to Ciprofloxacin and Other Antimicrobial Drugs in *Neisseria gonorrhoeae*, United States

**DOI:** 10.3201/eid1808.111202

**Authors:** Edward Goldstein, Robert D. Kirkcaldy, David Reshef, Stuart Berman, Hillard Weinstock, Pardis Sabeti, Carlos Del Rio, Geraldine Hall, Edward W. Hook, Marc Lipsitch

**Affiliations:** Harvard School of Public Health, Boston, Massachusetts, USA (E. Goldstein, M. Lipsitch);; Centers for Disease Control and Prevention, Atlanta, Georgia, USA (R.D. Kirkcaldy, S. Berman, H. Weinstock);; Oxford University, Oxford, UK (D. Reshef);; Harvard University, Cambridge, Massachusetts, USA (P. Sabeti);; Emory University, Atlanta (C. Del Rio);; Cleveland Clinic, Cleveland, Ohio, USA (G. Hall); University of Alabama at Birmingham, Birmingham, Alabama, USA (E.W. Hook);; and Jefferson County Department of Health, Birmingham (E.W. Hook)

Gonorrhea is the second most frequently reported communicable disease in the United States (*1*). Following implementation of a national gonorrhea control program in the mid-1970s, gonorrhea incidence in the Unites States declined by 74.3% from 1975 to 1997 (*2*). However, during this time, the treatment and control of gonorrhea have been complicated by the appearance and spread of antimicrobial drug resistance in *Neisseria gonorrhoeae* (*3**,**4*). Cephalosporins and fluoroquinolones became recommended for treating gonorrhea in the United States in 1993 (*5*), prompted by the rise in resistance to penicillins and tetracyclines, the antimicrobial agents previously recommended for treatment. In the past decade, several countries reported a sharp rise in the proportion of *N. gonorrhoeae* strains resistant to fluoroquinolones (QRNG) (*1**,**4**,**6**–**9*). Increasing QRNG prevalence in the United States led to a series of changes in treatment recommendations away from fluoroquinolones. In 2002, the Centers for Disease Control and Prevention (CDC; Atlanta, Georgia, USA) recommended that cephalosporins be used instead of fluoroquinolones as first-line treatment for gonorrhea acquired in Hawaii or California (*8*); in 2004, fluoroquinolones were no longer recommended as a first line of treatment for infected men who have sex with men (MSM) and, as of 2007, fluoroquinolones were no longer recommended, and cephalosporins were the only recommended class of drugs for treatment of gonococcal infections in the United States (*10*). As treatment failures for oral cephalosporins are documented in Asia (*3**,**11**,**12*), and strains with reduced susceptibility to cephalosporins have begun to appear in the West, including the United States (*3**,**13**,**14*), the origins and causes of increased drug resistance in *N. gonorrhoeae* need to be understood in order to improve control measures for emerging resistant strains and thereby maintain the utility of the few existing antimicrobial drug options for treatment of gonorrhea.

We examined several hypotheses to explain the increased prevalence of QRNG during 2002–2007, with the objective of identifying principles that may be informative for predicting and preventing the spread of resistance to cephalosporins or other drug classes. We considered the role of travel as a contributing factor for the growth of resistance, in heterosexual men and MSM. Having observed a difference in travel patterns between men of differing sexual orientation, we hypothesized about a potential role that multidrug resistance may play in the propagation of that resistance profile. Additionally, we studied the association between the times of first appearance of ciprofloxacin resistance in heterosexual men and in MSM in several different Gonococcal Isolate Surveillance Project (GISP) sites, examining the possibility that resistance spread in persons of one sexual orientation led to the appearance of resistance in persons of another sexual orientation.

## Methods

### Data

GISP is a national sentinel surveillance system for detection of gonococcal antimicrobial drug resistance that includes sexually transmitted disease (STD) clinics in 25–30 US cities each year, 4–5 regional laboratories, and CDC. Each month, the first 25 urethral gonococcal isolates were collected from men attending the clinics and submitted to regional laboratories for antimicrobial drug susceptibility testing. Patient data were abstracted from medical records.

Methods for *N. gonorrhoeae* susceptibility testing have been described (*15*). Briefly, Difco GC base medium (Becton Dickinson, Sparks, MD, USA) was inoculated with 10^4^ CFU. Antimicrobial drug susceptibilities were determined by the agar-dilution technique with GC-II base medium (Becton Dickinson). Control strains (F-18 [ATCC 49226], F-28, P681E, CDC 10328, CDC 10329, SPJ-15, and SPL-4) were included with each susceptibility run.

### Definitions

Sexual orientation is a standard GISP data element and is specified as follows: heterosexual men (82.9% of GISP patients during 1998–2007), MSM (13.8%), or bisexual men (3.4%). Temporal analyses of antimicrobial drug resistance, stratified by the infected person’s sexual orientation were largely restricted to heterosexual men and MSM. Travel history has been collected in GISP since mid-2002, with the aim of exploring whether acquisition of gonorrhea was local or occurred elsewhere. Until January 2004, travel history was defined as self-reported travel to Hawaii or outside the United States within the past 60 days; after that, travel history pertained to travel outside the state where the clinic was located. The above change was introduced because the aim of the travel variable was to assess the scope of nonlocal acquisition of gonorrhea. By 2004, QRNG was established in the United States, and it appeared reasonable to combine all out-of-state travel (both outside and within the United States) into the category of potential nonlocal acquisition.

A strain’s resistance to ciprofloxacin was defined as an MIC >1 μg/mL, per Clinical and Laboratory Standards Institute criteria (*16*). Temporal patterns of ciprofloxacin resistance among heterosexual men, MSM, and bisexual men are summarized in Figure 1. For strains resistant to penicillin and tetracycline, resistance was defined as an MIC >2 μg/mL. The term “triply resistant gonococci” was defined as isolates having resistance to ciprofloxacin, penicillin, and tetracycline. We use the word “type” to mean a defined pattern of susceptibility or resistance to the drugs of interest; by this definition, for penicillin, tetracycline, and ciprofloxacin, there are 2^3^ (8) different types.

**Figure 1:** Semiannual prevalence of resistance to ciprofloxacin in *Neissseria gonorrhoeae* isolates from the Gonococcal Isolate Surveillance Program for men who have sex with men (MSM), heterosexual men, and bisexual men.

### Prevalence of Resistance Phenotypes by Patient’s Sexual Orientation, Time, and Site

The proportion of isolates with various resistance phenotypes (sensitive or resistant to ciprofloxacin, penicillin, and tetracycline) was computed by half-year, separately for heterosexual men and MSM. Among GISP patients in each group who were infected with particular types, the proportion with a travel history was plotted for each half-year period.

### Travel History and Drug-Resistance Profiles

For certain gonococcal types (the triply resistant type and the monoresistant [ciprofloxacin resistant, tetracycline- and penicillin-sensitive] type), we studied the association between the presence of this type and the report of recent travel. We constructed 4 separate multivariate logic regression models: by sexual orientation of patient (MSM or heterosexual) and resistance type (multi- or single-resistant types); thus, 2 × 2 = 4 models. In each model (applied to all GISP specimens from persons with a given sexual orientation), the binary outcome *Y* is the presence of that resistance type (1 if the specimen is of that type), and the covariates are *M* (month of sample collection) and *RTH*_(_presence of recent travel history) (1 if travel history is present). Thus,

__

The regression coefficient *β_RTH_* is therefore the logarithm of the odds ratio for carrying a strain of the selected type for a person with recent travel history versus that of a person with no recent travel history. The regression coefficient *β_M_* is the logarithm of the odds ratio for carrying a strain of the selected type on month *M* + 1 versus month *M*. The latter can also be interpreted as the relative monthly growth rate for the selected type (*17*). Using the model in (*17*), suppose that the selected type grows exponentially at a monthly rate of *r*_1_, and the other types combined grow at a monthly rate *r*_2_ in the population. Thus the population presence of the selected type on month *M* is *c*_1_*exp*(*r*_1_ × *M*), and the presence of other types is *c*_2_*exp*(*r*_2_ × *M*). The probability that a random specimen on month *M* is of the selected type is therefore

__The odds ratio for carrying a strain of the selected type on month *M* + 1 versus month *M* is then *exp*(*r*_1_ – *r*_2_), where *r*_1_ – *r*_2_ is the relative growth rate for the selected type.

Because travel information was missing from some sites, we restricted the model to sites from which travel information was available for >50% of GISP patients. We conducted the regression analysis under the scenario of omitting the persons with missing travel information and through multiple imputations for those persons (*18*). Because missing values in various sites were largely found in consecutive temporal batches, we assumed that missing information was not correlated with presence of the chosen type or recent travel history during those periods at those sites. We imputed history of recent travel at random, using available data to calculate the proportion of GISP patients that were recent travelers by site and sexual orientation within a 1-year interval centered at the time of each missing sample. We performed 500 joint imputations, and the mean and variance of the regression coefficients were estimated as described (*18*, p. 86). The Hosmer and Lemeshow goodness-of-fit test was applied to the subset of the data for which travel information was available (>83% of data in each model).

Because data on travel history were lacking before mid-2002, the period we selected for the logistic regression analysis was October 2002–December 2006. Fifteen GISP sites, representing 64.6% of all MSM GISP patients during that period, had travel information on >50% of MSM patients (83.5% of patients from those sites). Sixteen GISP sites, representing 47.6% of all heterosexual GISP patients during that period, had travel information on >50% of heterosexual patients (89.8% of patients from those sites). For the monoresistant type of *N. gonorrhoeae* in MSM, the period selected was October 2002–December 2004 (by which point the growth in prevalence of that type appears to have stopped; Figure 2, panel A). Thirteen GISP sites, representing 45.9% of all MSM GISP patients during that period, had travel information for >50% of MSM patients (92.9% of patients from those sites).

**Figure 2:** Semiannual prevalence of the ciprofloxacin (Cipro)–resistant type of *Neisseria gonorrhoeae* for men who have sex with men (A) and heterosexual men (B), stratified by resistance characteristics to tetracycline (tet) and penicillin (pen).

### First Appearance and Persistence of Resistance in Heterosexual Men and MSM

We examined the time of first appearance of ciprofloxacin-resistant *N. gonorrhoeae* strains in heterosexual men and in MSM at GISP sites during 1998–2006. Only sites that reported continuously during those years were examined. Because low levels of ciprofloxacin resistance, particularly in strains from heterosexual men, were detected in the 1990s (and those are possibly attributable to different genetic lineages than those that caused the rise in resistance levels in the 2000s), we only looked at sites where resistance first appearance after 2000; thus, the first appearance of resistance in GISP was more likely to correspond to appearance of the new lineage at that site, rather than persistence of the old strain.

## Results

### Overall Patterns of Drug Resistance Stratified by Patient’s Sexual Orientation

Figure 1 shows the prevalence of ciprofloxacin resistance in GISP isolates during 1998–2007 for heterosexual men, MSM, and bisexual men. Resistance increased faster and reached higher levels among MSM (42.5% at peak) compared with patterns for heterosexual men (9.1% at peak), with resistance levels among bisexual patients being consistently somewhat lower than among MSM (peaking at 37.7%).

During the period of rapid expansion of fluoroquinolone resistance of *N. gonorrhoeae* among MSM, the type resistant to ciprofloxacin, tetracycline, and penicillin (hereafter, triply resistant) was the fastest growing class of ciprofloxacin-resistant types among MSM (Figure 2, panel A), reaching 25.6% of all MSM GISP specimens at the peak. The type resistant to ciprofloxacin and sensitive to tetracycline/penicillin (monoresistant type) also experienced substantial growth, on par with the type resistant to ciprofloxacin and tetracycline (peaking at 6.7% and 8.5%, respectively, for all MSM GISP specimens), though the growth in the prevalence of those 2 types appears to have stalled earlier than the growth in the prevalence of the triply resistant type.

Triple resistance has been the most common form of ciprofloxacin resistance since mid-2001 among heterosexual men (Figure 2, panel B), peaking at 3.8% of GISP specimens from heterosexual men, and it was followed closely by the monoresistant type, peaking at 3.6% of GISP specimens from heterosexual men.

### Recent Travel and Resistance among MSM and Heterosexual Men

To test the hypothesis that travel contributed to the increase in resistance to ciprofloxacin, we calculated the proportion of persons in the GISP dataset who reported recent travel (see Methods), stratified by the resistance pattern of their isolate and by patient’s sexual orientation (Figure 3).We performed multivariate logistic regression analysis to determine the association between particular types (triply resistant and monoresistant, for MSM and for heterosexual men), recent travel, and time. The Table gives the estimates of the regression coefficients (with 95% confidence bounds) for each choice of a resistance type and patient’s sexual orientation; interpretation of those coefficients is described in Methods.

**Figure 3:** Semiannual prevalence of recent travel history in different type carriers for men who have sex with men (A) and heterosexual men (B). Ciprofloxacin (Cipro) sensitive, MIC <0.0625 μm/L.

**Table:** Regression coefficients and the p values for 4 groups, according to resistance type and patient’s sexual orientation

Model*	*β*_0_ (95% CI)	*β_RTH_* (95% CI)	*β_M_* (95% CI)	Hosmer-Lemeshow p value
Men who have sex with men				
Triply resistant	−2.48 (−2.72 to −2.24)	−0.328 (−0.650 to 0.005)	0.03 (0.023 to 0.037)	0.19
Monoresistant	−3.75 (−4.42 to −3.08)	0.51 (−0.18 to 1.2)	0.046 (0.006 to 0.086)	0.18
Heterosexual men				
Triply resistant	−4.72 (−5.08 to −4.36)	1.17 (0.81 to1.53)	0.022 (0.011 to 0.033)	0.35
Monoresistant	−5.05 (−5.49 to −4.60)	0.543 (−0.02 to 1.11)	0.018 (0.005 to 0.032)	0.69

*Logistic model given by equation 1.

Overall, triple resistance prevalence in isolates infecting MSM grew rapidly despite negative association with recent travel (odds ratio [OR] 0.72 for a GISP specimen to be triply resistant from MSM with a recent travel history versus those without a recent travel history, 95% CI (0.52–0.99)), while travel contributed to the growth of triple resistance levels in heterosexual men (corresponding OR 3.22, 95% CI [2.24–4.62]). The positive association between monoresistance and recent travel suggested by Figure 3 was borderline statistically significant for heterosexual men (corresponding OR 1.72, 95% CI [0.98–3.03]) and not statistically significant for MSM.

### First Appearance of Resistance in Strains from Heterosexual Men and MSM

To assess the extent to which the ciprofloxacin resistant type may bridge (i.e., spread across from) populations that differ in sexual orientation, we compared the month of first appearance for ciprofloxacin resistance in heterosexual men and MSM (Figure 4). Sharing of the resistant strains would be expected to result in correlated timing of the appearance of the resistant type, with sites where resistance that appeared early in MSM also appeared early in heterosexual men. Assuming that sampling was identical for MSM and heterosexual men, then the ordering within a city would provide evidence about the possible route of transmission; however, different proportions of MSM and heterosexual men at each site mean that correlated timing is interpretable as evidence of transmission, but the direction of transmission is difficult to determine.

**Figure 4:** First month of appearance of ciprofloxacin resistance in *Neisseria gonorrhoeae* isolates from heterosexual men and from men who have sex with men (MSM). ALB, Albuquerque, NM; ATL, Atlanta, GA; BAL, Baltimore, MD; BHM, Birmingham, AL; CHI, Chicago, IL; CLE, Cleveland, OH; LBC, Long Beach, CA; MIA, Miami, FL; MIN, Minneapolis, MN; PHI, Philadelphia, PA; PHX, Phoenix, AZ; POR, Portland, OR.

The months of first appearance for ciprofloxacin resistance in 12 cities by patient’s sexual orientation are documented in Figure 4. The Spearman rank correlation coefficient for the times of first appearance among MSM and heterosexual men at different GISP sites is 0.79, p = 0.002. This finding suggests that for a pair of GISP sites A and B, if resistance in isolates from persons of one sexual orientation appeared earlier at site A than at site B, resistance in persons of another sexual orientation was also likely to appear earlier at site A than at site B.

## Discussion

A common explanation for the rise in QRNG is repeated importation (*9**,**19*); some evidence that importation played a role in the initial growth of resistance in the United Kingdom can be seen in data from the Gonococcal Resistance to Antimicrobials Surveillance Programme (*20*). However, genetic evidence for a combination of importation, followed by internal proliferation of fluoroquinolone resistance in *N. gonorrhoeae,* has been reported (*21**,**22*). Similarly, GISP data suggest that whereas importation may play a substantial role when resistance levels are relatively low, the prevalence of resistance for certain types increases further for certain types without the aid of importation. Indeed, for heterosexual men, for whom levels of ciprofloxacin resistance were relatively low, resistance of the GISP gonococcal isolates was positively associated with patient’s recent travel. Nevertheless, prevalence of QRNG was much higher in MSM, with the triply resistant type being the most prominent component in the rise of ciprofloxacin resistance in GISP isolates. Starting at the end of 2002, when GISP travel data became available and 7% of GISP isolates from MSM were triply resistant, prevalence levels for that type of isolate from MSM further increased along with negative association with recent travel. Given the lack of travel data before mid-2002, we cannot, however, exclude the possibility that the initial growth in the prevalence of the triply resistant type in MSM was aided by importation.

In addition, using ciprofloxacin for gonorrhea treatment may have contributed to the increase in ciprofloxacin resistance. Indeed, ciprofloxacin resistance has declined significantly since 2007 when CDC indicated that fluoroquinolones were no longer a recommended treatment for gonorrhea (*23*). At the same time, although CDC stopped recommending fluoroquinolones for gonorrhea treatment in California and Hawaii in 2002 and nationally for MSM in 2004, ciprofloxacin resistance continued to increase in California and among MSM for years after those recommendations were made. However, adherence to these recommendations might have been poor in non-GISP settings because the oral cephalosporins recommended for gonorrhea treatment were not marketed in the United States during the study period (*12*). Community usage of fluoroquinolones for other indications might have also contributed to the rise in ciprofloxacin resistance in *N. gonorrhoeae*. Fluoroquinolones were the most commonly prescribed class of antimicrobial agents in the United States around the study period (*24*).

The prominence of multidrug resistance, particularly among MSM, combined with the negative association with recent travel in MSM starting in late 2002, suggests that this type has proliferated without the aid of repeated importation. Resistance to a variety of antimicrobial agents for that type raises the possibility that usage of nonfluoroquinolone antimicrobial drugs could have played a role in propagation of multidrug resistance. Data reported by Kent et al. (*25*) show high rates of asymptomatic *N. gonorrhoeae* infection among MSM attending STD clinics in San Francisco. Some of those patients likely received antimicrobial drugs to treat conditions other than gonorrhea. For such persons, multidrug resistant *N. gonorrhoeae* strains are more likely to survive the antimicrobial drug treatment, giving the multidrug-resistant type a selective advantage over other *N. gonorrhoeae* types. Further studies are needed to assess the scope of antimicrobial drug use for various indications, including STD treatment, in asymptomatic *N. gonorrhoeae* infections and the magnitude of the possible selective advantage.

The presence of bisexual men in the GISP data suggests a possibility for passage of resistant strains between the MSM and heterosexual communities. To address this question, we explored whether associations could be shown between the time of first appearance of ciprofloxacin resistance in heterosexual men and in MSM at different GISP sites. The timing of first appearance is highly correlated between heterosexual men and MSM, with a median delay (in either direction) of 5.5 months, despite a 5-year range of times of first appearance across different sites. Moreover, this correlation was observed in different geographic regions at different times, suggesting that resistant strains spread from 1 group to the other within each city. Given that heterosexual men are much more numerous than MSM among the GISP patients, the fact that resistance was detected first in MSM in half of the cities may reflect the more rapid rate of increase in MSM than in heterosexual men (described previously) and possibly also that the resistant type truly appeared first in MSM in most cities but was first recorded in heterosexual men in some places; our data do not enable us to disentangle these 2 contributions.

Our study had several limitations. The use of travel data to infer the role of out-of-site acquisition of resistance is complicated by several factors: travel data go back only to mid-2002; until 2004 “travel” signified the presence of travel abroad or to Hawaii within the past 60 days, and later it was changed to include any out-of-state travel. Moreover, during the periods for which our logistic regression analysis was performed, travel data were missing for many GISP patients. For periods when travel data were missing, we note that our regression analysis included only clinics that had travel information available for >50% of patients of the selected sexual orientation. Moreover, multiple imputations were used to deal with the missing travel data (*18*). Of course, many gonorrhea cases are not captured by GISP, and travel by these infected persons may have been critical in the introduction of the resistant strains.

Although gonorrhea patients with recent travel history may not have acquired their infection during travel, the negative association between recent travel history and triple resistance argues against acquisition through travel as a driver of increasing prevalence of that type among MSM. Positive association with recent travel for other types argues that acquisition through travel played a role in the increase in resistance level of the corresponding type, although travel data do not enable us to quantify the extent of that role.

Our study on the rise of ciprofloxacin resistance sheds some light on the possible mechanisms that might contribute to the emergence of cephalosporin resistance. Importation of resistance and acquisition through domestic travel are likely to play a substantial role in the initial rise in resistance levels. Analysis of the rise in triple resistance levels in isolates infecting MSM suggests that resistance levels for some types, particularly the multidrug resistant type, may increase through selective advantage in domestic transmission. This increase is probably associated with antimicrobial drug usage; repeated importation is not required. Several studies (*13**,**14**,**26*) have shown that decreased *N. gonorrhoeae* susceptibility to cephalosporins such as cefotaxime is associated with resistance to other antimicrobial agents. This finding suggests that proliferation of multidrug resistance is also possible for cephalosporin-resistant *N. gonorrhoeae.* Correlation between appearance times in persons of the 2 sexual orientations further strengthens the argument that prevention efforts should be geared toward both groups, particularly when the appearance of resistance is detected in either population.

## References

[1] Centers for Disease Control and Prevention. Gonococcal Isolate Surveillance Project annual report. 2007 [cited 2012 May 22]. http://www.cdc.gov/std/gisp2007/GISPSurvSupp2007Complete.pdf

[2] Fox KK, Whittington WL, Levine WC, Moran JS, Zaidi AA, Nakashima AK. Gonorrhea in the United States, 1981–1996. Demographic and geographic trends. Sex Transm Dis. 1998;25:386–93. 10.1097/00007435-199808000-000119713920

[3] Barry PM, Klausner JD. The use of cephalosporins for gonorrhea: the impending problem of resistance. Expert Opin Pharmacother. 2009;10:555–77. 10.1517/1465656090273199319284360PMC2657229

[4] Workowski KA, Berman SM, Douglas JM Jr. Emerging antimicrobial resistance in *Neisseria gonorrhoeae*: urgent need to strengthen prevention strategies. Ann Intern Med. 2008;148:606–13.1841362210.7326/0003-4819-148-8-200804150-00005

[5] Centers for Disease Control and Prevention. 1993 sexually transmitted diseases treatment guidelines. MMWR Recomm Rep. 1993;42(RR-14):1–102.8145706

[6] Borgen K, van Loo I, Koedijk F, van de Laar M. Increase of gonococcal quinolone resistance in the Netherlands from 2002–2004. Euro Surveill. 2005;10:E051117.4. 1679428310.2807/esw.10.46.02836-en

[7] Fenton KA, Ison C, Johnson AP, Rudd E, Soltani M, Martin I, Ciprofloxacin resistance in *Neisseria gonorrhoeae* in England and Wales in 2002. Lancet. 2003;361:1867–9. 10.1016/S0140-6736(03)13489-712788575

[8] Centers for Disease Control and Prevention. Increases in fluoroquinolone-resistant *Neisseria gonorrhoeae*—Hawaii and California, 2001. MMWR Morb Mortal Wkly Rep. 2002;51:1041–4.12487525

[9] Tapsall JW, Limnios EA, Murphy D. Analysis of trends in antimicrobial resistance in *Neisseria gonorrhoeae* isolated in Australia, 1997–2006. J Antimicrob Chemother. 2008;61:150–5. 10.1093/jac/dkm43417986492

[10] Centers for Disease Control and Prevention. Update to CDC's sexually transmitted diseases treatment guidelines, 2006: fluoroquinolones no longer recommended for treatment of gonococcal infections. MMWR Morb Mortal Wkly Rep. 2007;56:332–6.17431378

[11] Tapsall JW. *Neisseria gonorrhoeae* and emerging resistance to extended spectrum cephalosporins. Curr Opin Infect Dis. 2009;22:87–91. 10.1097/QCO.0b013e328320a83619532086

[12] Newman LM, Moran JS, Workowski KA. Update on the management of gonorrhea in adults in the United States. Clin Infect Dis. 2007;44(Suppl 3):S84–101. 10.1086/51142217342672

[13] Lindberg R, Fredlund H, Nicholas R, Unemo M. *Neisseria gonorrhoeae* isolates with reduced susceptibility to cefixime and ceftriaxone: association with genetic polymorphisms in *penA, mtrR, porB1b*, and *ponA.* Antimicrob Agents Chemother. 2007;51:2117–22. 10.1128/AAC.01604-0617420216PMC1891421

[14] de Vries HJ, van der Helm JJ, Schim van der Loeff MF, van Dam AP. Multidrug-resistant *Neisseria gonorrhoeae* with reduced cefotaxime susceptibility is increasingly common in men who have sex with men, Amsterdam, the Netherlands. Euro Surveill. 2009;14:pii:19330. 1976173710.2807/ese.14.37.19330-en

[15] Schwarcz SK, Zenilman JM, Schnell D, Knapp JS, Hook EW III, Thompson S, National surveillance of antimicrobial resistance in *Neisseria gonorrhoeae*. The Gonococcal Isolate Surveillance Project. JAMA. 1990;264:1413–7. 10.1001/jama.1990.034501100590272144026

[16] Clinical and Laboratory Standards Institute. Performance standards for antimicrobial susceptibility testing, 2010. Wayne (PA): The Institute; 2010.

[17] Lipsitch M. The rise and fall of antimicrobial resistance. Trends Microbiol. 2001;9:438–44. 10.1016/S0966-842X(01)02130-811553456

[18] Little RJA, Rubin DB. Statistical analysis with missing data. 2nd ed. Hoboken (NJ): Wiley; 2002. p. xv.

[19] Tapsall J. Multidrug-resistant *Neisseria gonorrhoeae.* CMAJ. 2009;180:268–9. 10.1503/cmaj.08172119188616PMC2630337

[20] Health Protection Agency. The Gonococcal Resistance to Antimicrobials Surveillance Programme: annual report, 2007 [cited 2012 May 22]. http://www.hpa.org.uk/web/HPAwebFile/HPAweb_C/1221117895841

[21] Martin IM, Ison CA, Aanensen DM, Fenton KA, Spratt BG. Changing epidemiologic profile of quinolone-resistant *Neisseria gonorrhoeae* in London. J Infect Dis. 2005;192:1191–5. 10.1086/44442916136461

[22] Pérez-Losada M, Crandall KA, Bash MC, Dan M, Zenilman J, Viscidi RP. Distinguishing importation from diversification of quinolone-resistant *Neisseria gonorrhoeae* by molecular evolutionary analysis. BMC Evol Biol. 2007;7:84. 10.1186/1471-2148-7-8417543117PMC1906744

[23] Centers for Disease Control and Prevention. GISP profiles, 2009. [cited 2012 May 22]. http://www.cdc.gov/std/gisp2009/default.htm

[24] Linder JA, Huang ES, Steinman MA, Gonzales R, Stafford RS. Fluoroquinolone prescribing in the United States: 1995 to 2002. Am J Med. 2005;118:259–68. 10.1016/j.amjmed.2004.09.01515745724

[25] Kent CK, Chaw JK, Wong W, Liska S, Gibson S, Hubbard G, Prevalence of rectal, urethral, and pharyngeal chlamydia and gonorrhea detected in 2 clinical settings among men who have sex with men: San Francisco, California, 2003. Clin Infect Dis. 2005;41:67–74. 10.1086/43070415937765

[26] Tanaka M, Nakayama H, Huruya K, Konomi I, Irie S, Kanayama A, Analysis of mutations within multiple genes associated with resistance in a clinical isolate of *Neisseria gonorrhoeae* with reduced ceftriaxone susceptibility that shows a multidrug-resistant phenotype. Int J Antimicrob Agents. 2006;27:20–6. 10.1016/j.ijantimicag.2005.08.02116318912

# Research article: Factors Related to Increasing Prevalence of Resistance to Ciprofloxacin and Other Antimicrobial Drugs in *Neisseria gonorrhoeae*, United States

**DOI:** 10.3201/eid1808.111202

**Authors:** Edward Goldstein, Robert D. Kirkcaldy, David Reshef, Stuart Berman, Hillard Weinstock, Pardis Sabeti, Carlos Del Rio, Geraldine Hall, Edward W. Hook, Marc Lipsitch

**Affiliations:** Harvard School of Public Health, Boston, Massachusetts, USA (E. Goldstein, M. Lipsitch);; Centers for Disease Control and Prevention, Atlanta, Georgia, USA (R.D. Kirkcaldy, S. Berman, H. Weinstock);; Oxford University, Oxford, UK (D. Reshef);; Harvard University, Cambridge, Massachusetts, USA (P. Sabeti);; Emory University, Atlanta (C. Del Rio);; Cleveland Clinic, Cleveland, Ohio, USA (G. Hall); University of Alabama at Birmingham, Birmingham, Alabama, USA (E.W. Hook);; and Jefferson County Department of Health, Birmingham (E.W. Hook)

## Medscape CME Quiz

## Earning CME Credit

To obtain credit, you should first read the journal article. After reading the article, you should be able to answer the following, related, multiple-choice questions. To complete the questions (with a minimum 70% passing score) and earn continuing medical education (CME) credit, please go to www.medscape.org/journal/eid. Credit cannot be obtained for tests completed on paper, although you may use the worksheet below to keep a record of your answers. You must be a registered user on Medscape.org. If you are not registered on Medscape.org, please click on the New Users: Free Registration link on the left hand side of the website to register. Only one answer is correct for each question. Once you successfully answer all post-test questions you will be able to view and/or print your certificate. For questions regarding the content of this activity, contact the accredited provider, CME@medscape.net. For technical assistance, contact CME@webmd.net. American Medical Association’s Physician’s Recognition Award (AMA PRA) credits are accepted in the US as evidence of participation in CME activities. For further information on this award, please refer to http://www.ama-assn.org/ama/pub/category/2922.html. The AMA has determined that physicians not licensed in the US who participate in this CME activity are eligible for *AMA PRA Category 1 Credits*™. Through agreements that the AMA has made with agencies in some countries, AMA PRA credit may be acceptable as evidence of participation in CME activities. If you are not licensed in the US, please complete the questions online, print the certificate and present it to your national medical association for review.

## Article Title: Increasing Resistance to Ciprofloxacin and Other Antimicrobial Drugs in *Neisseria gonorrhoeae,* United States

## CME Questions

**1. You are a consultant to a US public health department regarding development of antibiotic resistance in Neisseria gonorrhoeae. Based on the analysis of data from the Gonococcal Isolate Surveillance Project (GISP) by Dr. Goldstein and colleagues, which of the following statements about overall patterns of drug resistance stratified by sexual orientation is most likely correct?**

A. Between 1998 and 2007, ciprofloxacin resistance grew faster for heterosexual men than in men who have sex with men (MSM)

B. The fastest growing class of ciprofloxacin-resistant types in MSM was the type resistant to ciprofloxacin, tetracycline, and penicillin (triply resistant)

C. The mono-resistant type (resistant to ciprofloxacin; sensitive to tetracycline/penicillin) declined overall during the study period

D. In heterosexuals, the mono-resistant type was the most common form of ciprofloxacin resistance since mid 2001

**2. Based on the analysis of data from GISP by Dr. Goldstein and colleagues, which of the following statements about recent travel and resistance in MSM and heterosexuals is most likely correct?**

A. In MSM, triple resistance prevalence was positively associated with recent travel

B. In heterosexuals, triple resistance prevalence was negatively associated with recent travel

C. The positive association between mono-resistance and recent travel was borderline statistically significant for heterosexuals

D. In MSM, there was a strong, statistically significant association between mono-resistance and recent travel

**3. Based on the analysis of data from GISP by Dr. Goldstein and colleagues, which of the following statements about the first appearance of resistance in heterosexuals and MSM would most likely be correct?**

A. The timing of first appearance of ciprofloxacin resistance in heterosexuals and in MSM in different GISP sites was not correlated

B. Median delay in timing of first appearance between MSMs and heterosexuals was 5.5 months in either direction, despite a 5-year range of times of first appearance across different sites

C. Correlations were limited to the same geographic regions at the same times

D. The data suggest that prevention efforts should target only MSM

## Activity Evaluation

**Table:** 

1. The activity supported the learning objectives.				
Strongly Disagree				Strongly Agree
1	2	3	4	5
2. The material was organized clearly for learning to occur.				
Strongly Disagree				Strongly Agree
1	2	3	4	5
3. The content learned from this activity will impact my practice.				
Strongly Disagree				Strongly Agree
1	2	3	4	5
4. The activity was presented objectively and free of commercial bias.				
Strongly Disagree				Strongly Agree
1	2	3	4	5
